# Analyzing the nexus between Chinese industrial policy and cross-border M&As

**DOI:** 10.1371/journal.pone.0299030

**Published:** 2024-05-02

**Authors:** Zhiyong Dong, Zhigan Zhu, Dawei Song, Hongyu An

**Affiliations:** 1 Business School, Taizhou University, Taizhou, China; 2 Wuhan Mental Health Center, Wuhan, China; 3 School of Finance, Anhui University of Finance and Economics, Bengbu, China; 4 School of Journalism and Communication, Shanghai International Studies University, Shanghai, China; Federal University of Goias: Universidade Federal de Goias, BRAZIL

## Abstract

In recent years, as China experiences economic expansion and its corporations become more global, it has notably become a central hub for cross-border mergers and acquisitions (M&A) on the world stage. The Chinese government, in tandem, leverages these international M&A operations to drive industrial transformation and progress in technology. This research investigates the role of China’s industrial policies in shaping cross-border M&A activities by examining recent instances. Findings indicate that relaxing financial barriers and applying specific industrial tactics bolster companies’ abilities to secure funding, consequently energizing cross-border M&A initiatives. Several firms in these international mergers and acquisitions are intricately connected to political strategies, markedly affecting the formulation of industrial policies. This assertion is corroborated through the analysis of relevant statistical evidence. The study methodically collects and scrutinizes data to quantitatively depict the current landscape and influencing elements of cross-border M&A, thus providing concrete evidence for policy and business strategy formulation.

## 1. Introduction

In the era of globalization, industrial policy has become increasingly important in shaping national economic frameworks and guiding the actions of enterprises. Governments are increasingly turning to industrial policy [1] to strengthen the competitiveness and innovation of their industries. As the second largest economy in the world, the importance of China’s industrial policy is self-evident [2]. Since its reform and opening up, China has successfully attracted a large amount of foreign direct investment (FDI) through a series of policies and measures, which has promoted the rapid development of domestic industries [3]. According to China Venture Capital (CVC), the number of cross-border M&A in China over the past decade is 25 times higher than that of the ten-year period from 1995 to 2004, while the amount of cross-border M&A has increased 135 times. The average value of each international M&A deal by Chinese companies is about $320 million, which is 8.4 times the average of domestic M&A deals. Over the past decade, the number of overseas M&A deals by Chinese enterprises has grown at a compound annual growth rate of 45.89%, and the value of the deals has grown at an average annual rate of 15.54%. This rate far exceeds that of domestic M&A, highlighting the remarkable and rapid expansion of Chinese enterprises in the field of international M&A. In the context of globalization, cross-border M&A has become an important way for enterprises in various countries to expand their market share and acquire resources and technologies [4].

Previous research works have explored the impact of industrial policies on cross-border M&As in the institutional context of China and have made some key points. These studies point out that the policy orientation and institutional environment of the Chinese government have an important impact on firms’ M&A decisions [5] and emphasize the guiding role of industrial policy in cross-border M&A by Chinese firms [6, 7]. China’s industrial policies are usually oriented to encourage and support specific industries or fields, and promote cross-border M&As by providing financial support, tax breaks, and market access [8]. However, although studies have pointed out that the Chinese government’s policy orientation and institutional environment have an impact on firms’ M&A decisions [9], the specific influence mechanisms and effect paths still need to be further explored.

Based on the above analysis, the main objectives of this study are: (i) To explore the impact of industrial policies formulated by the Chinese government on the number and scale of cross-border M&As. The effectiveness of the Chinese Government’s industrial policies in promoting cross-border M&As is explored in depth. (ii) Analyze the role of Chinese SOEs in cross-border M&A activities and examine the extent to which government industrial policy guides and supports the participation and influence of these firms. (iii) Explore the M&A premium in cross-border M&A for target firms supported by industrial policies.

## 2 Literature review

In recent years, governments around the world have adopted various forms of industrial policies aimed at actively promoting economic development and enhancing national competitiveness [10]. These industrial policies have attracted widespread attention in the field of economics and policy research, and studies have shown that the implementation of these policies has a positive impact on enterprises [11]. Such financial assistance can significantly increase the ability of firms to withstand risks and enhance their stability and flexibility under conditions of economic uncertainty [12]. In addition, industrial policies can also stimulate the M&A activities of enterprises, thus further enhancing their competitiveness. Currently, China is a notable case in the field of global industrial policy. The Chinese government has actively formulated and implemented a series of industrial policies that have had a profound impact on the global expansion and international strategies of domestic firms [13, 14]. As the global economy continues to evolve, the importance and influence of industrial policy will continue to grow.

The idea that cross-border M&A decisions and outcomes are influenced by a variety of factors is widely recognized [15–21]. Economic considerations, cross-border M&As enable firms to acquire advanced technology and management experience [22, 23], integrate resources to expand market share, and enhance competitiveness. In addition, the economy of scale effect can reduce enterprise costs. Political and legal factors, such as national tax policies, also have an impact on business decisions [16, 20]. Cultural differences may increase integration barriers [15], so firms need to focus on cultural fit. Internal factors, such as strategic goals and resource strength, determine the direction of firms’ activities [17, 18]. At the same time, uncertainties in the political and economic environment, such as volatility, pose risks to firms’ activities [24–27]. Overall, variables such as changes in the political and economic climate introduce risk factors to these operations. One study found that state-owned enterprises (SOEs) usually have an advantage in cross-border M&As, compared to the lower position of Chinese private enterprises [28]. Meanwhile, SOEs have higher legitimacy in foreign markets, while lack of legitimacy [29] may lead to lower M&A performance.

Currently, there are relatively few studies analyzing the relationship between industrial policy and cross-border M&A, and only a few studies have used domestic data from the United States or China to study the impact of economic policy uncertainty on corporate M&A activities [4, 30, 31]. It has been suggested that cross-border M&As bring a greater degree of benefits to home country firms, such as development through the elimination of trade barriers, the exertion of greater market power, and the realization of a higher level of cross-integration of new knowledge and resources [32]. The timing, direction and content of economic policy adjustments in the host country can have an impact on the decision-making behavior and economic consequences for economic entities [33]. When the economic environment fluctuates dramatically, the government formulates or changes economic policies to steer the direction of economic development [34]. However, the research on the detailed relationship between industrial policy and cross-border M&A and its influencing factors still needs to be further explored in depth.

## 3 Hypothesis development

Political shocks are fundamental political risk factors faced by firms, which affect their strategic choices and business development in international markets [35–37]. In this context, cross-border mergers and acquisitions (M&A) as a strategic choice may be affected by government policies, including government support or restrictions on specific industries and market access conditions. Scholars have examined how trade policies affect the incentives for cross-border M&As. It has been found that changes in trade policies affect firms’ internationalization strategies and cross-border M&A decisions [38]. If the government adjusts trade policies to support cross-border M&A activities, firms may be encouraged and increase the number and scale of cross-border M&As. For example, the Chinese government has provided financial support and relaxed institutional restrictions for MNCs [9]. China’s "going out" strategy encourages domestic enterprises to participate in the international capital market and make direct outward investments. This helps Chinese firms to succeed in the internationalization process [8]. Therefore, we propose hypothesis 1.

Hypothesis 1: The industrial policy formulated by the Chinese government has a certain role in promoting the number and scale of cross-border M&As.

Recent studies have begun to explore how institutional-level and firm-level factors affect the number and type of cross-border M&As [39, 40]. In their study, Stearns et al. make the point that changes in the economic and political environment can create conditions conducive to a wave of M&As [41]. Particularly in Chinese state-owned enterprises, political capital exists and has an impact on corporate activities [42]. In addition, it has also been pointed out that the effect of institutions on firms’ cross-border M&A performance depends on the type of firms [43]. For Chinese SOEs, government industrial policies can provide a guiding framework and supportive measures to provide better conditions and resources for cross-border M&A of SOEs, which can help them cope with challenges from different countries. Currently, SOEs are becoming more and more competitive and are beginning to exhibit more diverse strategies [44]. Considering that SOEs, as a special type of enterprises, face unique institutional environments and requirements in cross-border M&As, SOEs are usually more likely to break through industry barriers than non-SOEs [45], providing more favorable conditions and support for SOEs to improve their cross-border M&A performance. Therefore, we propose hypothesis 2.

Hypothesis 2:The participation and influence of Chinese SOEs in cross-border M&A is guided and supported by the government’s industrial policy.

Some studies have found that target firms supported by industrial policies may have relative advantages in terms of technology, market share or patents [46]. Such policy support can increase investors’ confidence in the target firm, which makes them more willing to pay a higher M&A premium to gain control of the firm [16]. Industrial policy support provides the target firm with an advantage in the competitive market. For example, policy support reduces the entry of competitors and provides the target firm with more government subsidies and bank loans [47]. These policy supports help to enhance the competitiveness and value of the target firm, making it an attractive M&A target. As a buyer firm may be willing to pay a higher M&A premium in order to obtain these advantages [48]. At the same time, target firms supported by industrial policies may face lower risks relative to other firms. The process of cross-border M&A involves certain risks, such as cultural differences [15], legal and regulatory uncertainties [16, 20], and market challenges [49]. However, policy support can provide certain guarantees for buyer firms, and this reduced risk may lead to buyer firms being more willing to pay a higher M&A premium. Therefore, we propose hypothesis 3

Hypothesis 3: Target firms supported by industrial policies will receive higher M&A premiums in cross-border M&A.

## 4 Standards and processes

### 4.1 Parameters of the model

#### 4.1.1 Benchmark design

In this section, the theoretical approach to analyze the influence of industrial policy on international M&A decisions by firms is introduced, followed by an empirical investigation. To bolster the credibility of the findings and address the issue of non-time-varying missing variables, the study adopts panel data analysis techniques [50]. The application of panel data helps to address the heterogeneity between industrial policies and firm choices and to analyze them on the basis of a large sample size. By tracking data from the same firms across time and place, the study is able to accurately measure the impact of industrial policy on cross-border M&A choices.

The specific model is as follows:

$$cbma_{it}=a_{0}+a_{1}policy_{it}+a_{2}X_{it}+\mu_{i}+\gamma_{t}+\varepsilon_{it}$$ (1)

The variable *cbma*_*it*_ is used to measure whether a firm has successfully implemented a cross-border M&A, where subscript t represents a time series and subscript i represents the different companies involved. *cbma*_*it*_ is set to 1 for successful implementation of cross-border M&A and 0 for unsuccessful implementation. On the other hand, the binary variable, "policy", is used to indicate whether the industry in which the listed company operates is supported by industrial policy, and it is set to 1 to indicate that it is supported, and it is set to 0 to indicate that it is not supported. x denotes the variable of the low-carbon economic transition used for control. Individual and time fixed effects are denoted by the symbols μ and γ, respectively. The random error term ε symbolizes unpredictable variations not accounted for by the model, while the policy coefficient serves as the explanatory variable. A positive value of this coefficient suggests a positive correlation between industrial policy and international M&A activities, while the negative coefficient indicates that the two are negatively correlated, reflecting that policy changes may affect the trend of international M&A.

Dynamic panel regression method was chosen to test robustness. The use of panel data allows us to better capture the relationship between an individual’s current behavior and past behavior [51]. Due to the existence of inertia in individual behavior, current behavior is often influenced by past behavior. In order to more fully consider the possible dynamic adjustments and inertia of firms’ M&A premiums, this study employs a dynamic panel model for robustness testing. This model not only allows for the lagged value of the dependent variable to be taken into account, but also more accurately reflects the time dynamics of individual behavior. Through the dynamic panel model, we are expected to gain a deeper understanding of the evolution of corporate M&A premiums, revealing the possible dynamic adjustment mechanisms and the effects of long-term inertia. The following are the relevant formulas:

$${CBMA}_{it}=\alpha_{0}+\rho {CBMApre}_{it-1}+\alpha_{1}{policy}_{it}+\alpha_{2}X_{it}+\mu_{i}+\gamma_{t}+\varepsilon_{it}$$ (2)

Due to the introduction of lags of the explanatory variables, in order to obtain consistent and efficient estimation results, we choose the generalized method of moments estimation, of which three commonly used methods are difference GMM, level GMM, and system GMM. The adoption of these methods aims to deal with the challenges of individual effects, lagging problems, and endogeneity that exist in panel data. First, difference GMM eliminates individual effects through first-order differencing, while introducing the difference term lagged one period behind the explanatory variables as an instrumental variable, which effectively solves the endogeneity problem caused by individual heterogeneity. Second, the level GMM approach directly uses the one-period lagged explanatory variable’s one-period difference term as an instrumental variable, which avoids the introduction of the first-order difference and makes the model more flexible. Finally, the systematic GMM approach is able to control both individual and temporal heterogeneity, and capture the effects of these heterogeneity by introducing hysteresis and differential variables. Compared with the differential GMM method, the system GMM method uses more information of panel data and improves the efficiency and accuracy of estimation. Compared with the horizontal GMM method, the system GMM method can deal with the endogeneity problem in the panel data model, and eliminates the estimation bias caused by endogeneity by using the lag variable as the instrument variable. Therefore, we choose the systematic GMM method for panel data analysis to effectively control the effects of endogeneity and heterogeneity [52].

#### 4.1.2 Inspection model of impact mechanism

In the context of cross-border mergers and acquisitions (M&A), the decision-making outcome is depicted using a binary variable, CBMA, with a value of 1 indicating a successful transaction and 0 signifying a failure. Simultaneously, an additional variable named "Policy" is introduced to assess whether the industry of the listed company receives support from industrial policies. In this model, a value of 1 signifies support, while 0 indicates its absence, providing a metric for researchers to analyze the effect of industrial policies on decision-making. Additionally, to factor in the influence of the shift towards a low-carbon economy, a control variable X is incorporated. Time and firm are denoted by subscripts t and i, respectively. Individual and time fixed effects are represented by μ and γ symbols, with ε symbolizing the stochastic error term that varies over time. As for the policy variable’s coefficient, its positive value implies that industrial policy favorably impacts cross-border mergers and acquisitions, whereas a negative value suggests a detrimental effect.


$$cbma_{it}=\alpha_{0}+\alpha_{1}policy_{it}+\alpha_{2}X_{it}+\mu_{i}+\gamma_{t}+\varepsilon_{it}$$ (3)

$$long\_asset_{it}=\alpha_{0}+\beta_{1}policy_{it}+\beta_{2}X_{it}+\mu_{i}+\gamma_{t}+\varepsilon_{it}$$ (4)

$$cbma_{it}=\alpha_{0}+\lambda_{1}policy_{it}+\lambda_{2}long\_asset_{it}+\lambda_{3}X_{it}+\mu_{i}+\gamma_{t}+\varepsilon_{it}$$ (5)

In Formula (4), the term "long assets" is incorporated as an explanatory variable to assess the financing capacity of each firm. The study employs a stepwise regression approach to methodically uncover the connection between cross-border M&A, China’s industrial policy, and corporate financing limitations. Initially, the model’s procedure is rendered clear and practical by sequentially introducing and testing explanatory variables. During the significance testing phase, an insignificant α1 suggests a tenuous link between cross-border M&A and China’s industrial policy, leading to a halt in testing for intermediation effects. Similarly, if β1 lacks significance, it implies a feeble association between industrial policy and corporate financial constraints, prompting an early discontinuation of the intermediary effect analysis. This methodology concentrates on pivotal variables, thereby enhancing the precision of the research.

Considering the influence mechanism of political relevance, this study uses firm characteristics to quantify political relevance. This not only increases the concreteness and operability of the model, but also improves the explanatory power of the research at the political level. The specific formula is as follows:

$$cbma_{it}=\alpha_{0}+\alpha_{1}policy_{it}+\alpha_{2}soe_{it}+\alpha_{3}policy_{it}*soe_{it}+\alpha_{4}X_{it}+\mu_{i}+\gamma_{t}+\varepsilon_{it}$$ (6)

In Eq (6), the "soe" variable stands for policy relevance and acts as a binary indicator. For state-owned enterprises, "soe" is set to 0, whereas for private enterprises, it is set to 1. The research emphasizes the coefficient of policy interaction with SOEs, representing the combined effect of policy and state ownership. A statistically significant and positive coefficient for the policy-state-owned enterprise interaction suggests that industrial policy substantially favors the cross-border M&A decisions of state-owned enterprises. A positive coefficient highlights that state-owned enterprises are more likely to undertake cross-border M&As under the guidance of industrial policies. This underscores the direct, positive influence of such policies on state-owned enterprises, prompting them to engage more actively in cross-border M&A activities.

#### 4.1.3 Merger premium model

This study develops a multifaceted regression analysis (1) to examine how industrial policy backing of M&A target companies influences the M&A premium:

$$premium_{i,t}=\alpha_{0}+\alpha_{1}policy+\alpha_{2}X_{it}+\mu_{i}+\gamma_{i}+\varepsilon_{i}$$ (7)

Variable *premium*_*i*,*t*_ is used to measure firms’ M&A premium, where subscript t represents the time series and subscript i represents the different firms involved. This paper uses OLS for estimation and controls for industry (Ind) and year (Year) fixed effects, and the t-values of the regression results are all t-values calculated with robust standard errors.

### 2.1.4 Variable definition

*(1) Dependent variable*. The decision of transnational merger of listed companies is the dependent variable of the analysis, and the symbol CBMA represents the variable. This metric indicates the occurrence of cross-border mergers by a listed company within a given year. To be precise, the CBMA value is set to 1 when the listed company engages in a cross-border merger in that year. Conversely, the CBMA value is recorded as 0 if the listed company does not undertake such a merger. In the context of this research, a transnational merger is defined as the acquisition of foreign enterprises by listed companies, achieved through purchasing equity or assets.

*(2) Explanatory variables*. Industrial policy: As the main explanatory variable. Industrial policy support is a one-two value variable, and the value 1 indicates that the listed company has obtained industrial support policy support; The value 0 indicates that the system is not supported.

The purpose of industrial policy support is to ascertain if there are any operational policies in place that influence the functioning of listed companies. These policies consist of a variety of government-devised strategies and initiatives aimed at steering and advancing the country’s economic growth.

Financial Limitations: The capacity of businesses to secure funding is employed as a representation of their financial constraints. To quantitatively measure this financing capacity, the study uses two key indicators, long-term borrowing capacity and corporate asset ratios, which are uniformly labeled as "Long Asset b".

Political relevance: The ownership structure of a company is used as a proxy for its political relevance. Use binary variables to represent the ownership structure of the company. The binary variable is labeled "soe" and takes a value of 1 or 0. The "soe" value is set to 1 for state-owned enterprises. On the other hand, non-state-owned companies are assigned a "soe" value of 0.

Control Variables: This encompasses a range of factors including the scale of the company, the ratio of assets to liabilities, asset returns, dual market presence, magnitude of sales, proportion of wages, age of the company, equity returns, rate of cost increase, and the price-to-earnings ratio. The incorporation of these variables is intended to mitigate the effects of extraneous elements on decisions regarding cross-border mergers and acquisitions, thereby enabling a more precise evaluation of how particular factors (like policies) impact these decisions.

Table 1 presents the definition of each variable used in the analysis.

**Table 1:** Variable description.

	Variable representation	The calculation method
Industrial policy	Policy	Compiled in accordance with the work report of the government
Cross-border acquisition of virtual variables	CBMA	SDC Platinum after ending claims SDC Platinum after ending claims
Merger and acquisition premium	P	(M&A consideration—book value of the target enterprise) / book value of the target enterprise
The size of the listed company	Size	Take a balance of the total assets of the listed company
Returns on listed companies’ net assets	ROE	Net income / total company assets
The relationship between corporate assets and liabilities	AD	Total liabilities/total assets
Return on Assets	ROA	Net profit/total assets
The number or size of board members	BD	Count the number of board members.
Listed companies are one of two jobs	Dual	If a listed company is a virtual variable
The principal revenue of a publicly traded company	IS	Take a balance of the major revenues of companies listed on the stock exchange under the profit statement
The pace of primary revenue growth for publicly traded companies	SG	By deducting the difference between the two, a listed company’s primary revenue is calculated
The percentage of listed companies’ managerial salaries	SR	The amount of management remuneration paid by publicly traded corporations, according to the literature, indicates how overconfident management is
How long a listed company has been in business	FA	How long a listed company has been in business
Listed companies’ equity return	ROE	Net income/net assets of publicly traded companies
Company cost increase	CG	The amount of the cost disparity between the listed company’s primary costs
Price-to-earnings ratio	PE	Share price to net assets ratio of a publicly traded company
The properties of listed companies	SOE	Whether a listed company is a virtual variable of a state-owned enterprise

*(3) Data collection*. The research object of this study is Chinese A-share companies listed in Shanghai and Shenzhen during the period from 2012 to 2022, aiming to explore in depth the mechanism of the influence of industrial policies on the cross-border M&A decisions of these companies. Table 2 showed the summary statistics for main variables. To ensure the validity of the research results, we conducted several rounds of screening on the initial sample. First, the financial sector sample was excluded to ensure that the research focus is more concentrated on real industries. Second, listed companies designated as ST were excluded to exclude samples that might be affected by special circumstances such as financial distress. Finally, data with missing values were cleaned. The study uses cross-border M&A transaction data provided by the SDC Platinum M&A Transaction Database, which has relatively complete historical data and rich variable information, providing us with the basis to dig deeper into the cross-border M&A behaviors of enterprises. Industrial policy data, on the other hand, is obtained through manual collection to ensure its accuracy and timeliness. Firm-level financial statistics are obtained from Cathay Pacific’s CSMAR database, which provides information support for the study in the financial dimension. In terms of data processing, to minimize the impact of outliers on the empirical results, we pruned the range of values of all continuous variables, limiting them to between 1% and 99%.

**Table 2:** Summary statistics for the main variables.

variable	Mean	Sd	min	max	N
CBMA	0.0677	0.1955	0	1	27400.00
Policy	0.4230	0.5582	0	1	27,400.00
P	4.7098	8.2048	-0.5422	1.4620	27,400.00
Size	26.7167	1.9709	13.2191	21.5993	25,700.00
AD	0.5485	0.4799	0.0123	69.8805	25,700.00
ROA	0.0529	0.0948	-5.4929	3.2556	25,700.00
Dual	0.9709	0.5062	0	1	24,600.00
IS	27.0237	1.7596	12.3673	31.5251	25,600.00
SG	0.8944	101.6385	-1.1799	15007.59	25,700.00
SR	0.5398	0.1129	0	1	27,400.00
FA	17.4962	4.9347	3	56	27,400.00
ROE	0.0484	0.4740	-32.83085	2576.40	27,400.00
CG	0.3659	7475	-1.008485	668.2820	25,400.00
PE	131.1623	2935.63	0.3863	382200.00	23,100.00

## 5 Results and discussion

### 5.1 Baseline analysis

The bidirectional fixed effects regression analysis method based on module is studied. By introducing individual fixed effects and time fixed effects, the data of different modules are analyzed to control possible confounding factors [53]. Two-way fixed effect model plays an important role in modular based regression analysis. It controls possible individual and time dependent effects in the model by introducing individual fixed effects and time fixed effects. This improves the reliability and robustness of the results.

According to columns 1 and 2 of Table 3, the coefficients of industrial policy are 0.030 and 0.026, respectively. The effect of industrial policy remains significant when other control variables are considered [33]. Some other control variables such as firm size, gearing ratio, ROA (return on total assets), dual attributes (representing SOEs and non-SOEs), natural logarithm of sales, sales growth rate, salary ratio, firm age, ROE (return on net assets), cost growth rate, and price-earnings ratio are also listed in the table.

**Table 3:** The influence of industrial policy on international merger and acquisition decision-making.

	(1) CBMA	(2) CBMA	(3) CBMA	(4) CBMA
Policy	0.029***	0.026***	0.024***	0.029**
	-8.586	-8.763	-1.701	-2.138
Size		0.013***		0.027***
		-4.185		-2.916
AD		-0.014		0.105***
		(-1.498)		-34.340
ROA		0.219***		0.013***
		-129.360		-254.410
Dual		-0.019		-0.016***
		(-5.326)		(-1.997)
IS		-0.005***		-0.020***
		(-1.279)		(-2.260)
SG		0.000		0.000
		(-2.423)		-1.992
SR		-0.000**		0.011
		(-234)		-0.694
FA		-0.001***		0.014
		(-3.753)		-0.952
ROE		-0.059**		-0.041
		(-2.107)		(-1.216)
CG		0.003**		0.001**
		-2.795		-2.618
PE		0.000		0.000
		-0.015		-0.006
Effect of individual fixation	NO	NO	YES	YES
The Effect of Time Fixation	NO	NO	YES	YES
_cons	0.058***	-0.038	0.062***	-0.331
	(26.533)	(-1.044)	(1395)	(-1.173)
R2	0.003	0.008	0.233	0.253
N	27445	22416	25142	21367

z statistics in brackets

* p < 0.1

** p < 0.05

*** p < 0.01

To strengthen the robustness of the results, we introduce additional control variables. Columns 3 and 4 of Table 3 consider individual fixed effects and time fixed effects, respectively. The coefficients obtained are 0.021 and 0.022, respectively, indicating that industrial policy still has a significant impact on low capitalization listed companies’ participation in cross-border M&A decisions. These results remain consistent and robust even after accounting for other influences and introducing control variables. The introduction of two-way fixed effects further enhances the validity of the results. The empirical evidence provided in this study supports Hypothesis 1, which states that the Chinese government’s industrial policy has a significant role in promoting the number and size of cross-border M&As.

### 5.2 Impact mechanism test

#### 5.2.1 Test of the Financial Constraint Mechanism (FCM)

Utilizing a stepwise regression methodology based on Eqs (1) and (3), this study aims to provide insight into the ways in which industrial policy can alleviate financing constraints in facilitating cross-border M&A. The data in Table 4 are obtained from Cathay Pacific’s CSMAR database, which provides firm-level financial statistics. We use specific indicators and variables from the CSMAR database to measure firms’ ability to obtain financing. The CSMAR database is one of the leading financial and accounting databases in China and is widely used in academic research and business analysis.

**Table 4:** Capacity for securing financing for businesses.

	(1) Long_Asset	(2) Long_Asset	(3) Long_Asset	(4) Long_Asset	(5) CMBA	(6) CMBA
Policy	0.007***	0.001*	0.006***	0.006***		0.015*
	-6.923	-2.205	-3.248	-295.120		-1.420
long_asset					0.030***	0.015***
					-2.000	-2.287
Size		0.035***		0.023***	0.023***	0.022***
		-56.704		-30.638	-3.179	-3.066
AD		0.169***		0.160***	0.055**	0.070**
		-60.931		-38.650	-3.169	-2.604
ROA		0.120***		0.035*	0.318***	0.266***
		-944.280		-2.135	-3.556	-2.531
Dual		0.012***		0.002	-0.011**	-0.012**
		-10.537		-2.011	(-1.481)	(-2.242)
IS		-0.050***		-0.021***	-0.015**	-0.011**
		(-44.921)		(-16.551)	(-3.026)	(-1.909)
SG		0		0	-0.000*	-0.000*
		-0.673		-1.180	(-1.381)	(-1.941)
SR		0.012**		0.003	0.014	0.011
		-1.941		-0.506	-0.647	-0.709
FA		-0.001***		-0.014***	0.021	0.016
		(-3.945)		(-3.785)	-0.989	-1.169
Roe		-0.023**		-0.027***	-0.026	-0.030
		(-2.088)		(-2.413)	(-0.874)	(-0.645)
CG		0		0.000	0.001**	0.001**
		(-0.601)		(-0.876)	-1.864	-1.626
Pe		0		0	0.000	0.001**
		(-0.466)		-0.157	(-0.064)	(-0.084)
Individual fixation effect	NO	NO	YES	YES	YES	YES
Time fixation effect	NO	NO	YES	YES	YES	YES
_cons	0.061***	-0.226***	0.060***	-0.099*	-0.490*	-0.468*
	(75.716)	(-22.291)	(58.458)	(-1.826)	(-1.759)	(-1.679)
R2	0.003	0.342	0.658	0.746	0.259	0.259
N	21543	18687	22571	18921	18370	18737

z data enclosed in brackets

* p < 0.1

** p < 0.05

*** p < 0.01

This study uses regression analysis to explore the effects of long-term assets, industrial policy support and mergers and acquisitions on firms’ financing ability. The results show (the first column of Table 4) that there is a significant positive correlation between long-term assets and the financing ability of enterprises (0.007***), that is, the increase of long-term assets will improve the financing ability of enterprises. Even when controlling for other variables (Column 4), the effect of long-term assets remains positive and significant (0.006***). In addition, the degree of industrial policy support is also positively correlated with firm financing ability (0.001*, second column), and this relationship remains unchanged after controlling for other variables (fourth column) (0.006***). We further add the enterprise merger and acquisition variable (CMBA), and the results show (Column 5) that cmba variable is positively correlated with financing ability (0.015*), indicating that M&A is conducive to improving financing ability. This positive correlation persists when controlling for other variables (column 6) (0.015*).

According to the results of the study, when planning capital budgets and business strategies, firms should consider increasing investment in long-term assets to improve their financing ability. A favorable industrial policy environment can facilitate enterprises’ access to financing and cross-border M&A activities [54]. These findings provide guidance and decision-making basis for enterprises. Enterprises can enhance their financing capacity and further promote their business development and internationalization strategies by increasing investment in long-term assets and actively responding to industrial policies. In addition, governments and relevant institutions should continue to formulate and implement policies that are conducive to enterprise financing and cross-border M&A, so as to create a better development environment for enterprises.

#### 5.2.2 The mechanism of industry political relevance

The purpose of this study is to explore how corporate political connections affect China’s industrial policy and the decision-making process of cross-border mergers and acquisitions, and to conduct empirical analysis using regression models. Table 5 showed the mechanism of political relevance on industrial policy and cross-border M&A decision-making. Specifically, we built multiple regression models to analyze the data. The model setup includes control variables such as firm size, return on assets and firm lifetime. The results of each model show that the political relevance of firms has a positive impact on the choice of cross-border M&A, and the correlation coefficients are 0.039, 0.022, 0.028 and 0.040, respectively, all of which are statistically significant. In addition, the analysis also includes variables related to state-owned enterprises. The results show that state-owned enterprises significantly affect the choice of cross-border mergers and acquisitions, and the coefficients are -0.016 and -0.025, both of which are statistically significant. In addition, the interaction between political affiliation and state of state-owned enterprises is also included in the analysis. The results show that this interaction significantly affects cross-border M&A decisions, with an increasing effect across model Settings (0.010, 0.020, 0.016, and 0.025), suggesting that soes’ influence on cross-border M&A choices may be amplified in a policy-driven environment [8].

**Table 5:** Mechanism of political relevance on industrial policy and cross-border M&A decision-making.

	(1)CMBA	(2) CMBA	(3) CMBA	(4) CMBA
Policy	0.039***	0.022***	0.028**	0.040**
	-6.843	-3.995	-1.882	-2.861
SOE	-0.016***	-0.025***		
	(-121)	(-839)		
c.policy#c.soe	0.010***	0.020***	0.016***	0.025***
	-3.308	-2.758	-4.109	-369.720
Size		0.010***		0.023***
		-144		-3.952
AD		-0.010		0.094***
		(-0.687)		-26.600
ROA		0.286***		0.412***
		-4.176		-301.300
Dual		-0.014***		-0.019***
		(-3.941)		(-3.120)
IS		-0.002		-0.015***
		(-1.083)		(-2.896)
SG		-0.000**		-0.000*
		(-2.237)		(-1.400)
SR		-0.080***		0.009
		(-818)		-0.415
FA		-0.001***		0.013
		(-2.901)		-1.065
ROE		-0.095**		-0.046
		(-2.882)		(-1.251)
CG		0.002***		0.001**
		-2.671		-3.085
pE		0.000		0.000
		(-0.015)		(-0.010)
Individual fixation effect	NO	NO	YES	YES
Time fixation effect	NO	NO	YES	YES
_cons	0.066***	-0.076**	0.061***	-0.325
	(22.391)	(-2.078)	(1145)	(-1.152)
R2	0.005	0.009	0.301	0.213
N	24361	22626	25711	20421

z statistics in parentheses

* p < 0.1

** p < 0.05

*** p < 0.01

The results of the study show that China’s industrial policy has a significant positive impact on cross-border M&A decisions, and this empirical evidence supports Hypothesis 2. With policy support, state-owned enterprises (SOEs) have assumed greater political responsibility and have become a key player in facilitating international M&A [10]. In order to strengthen the reliability of the study, the research methodology uses a two-way fixed effects model and incorporates a series of control variables. The results provide valuable insights into the decision-making process of policymakers and corporate executives.

#### 5.2.3 Regression analysis of industrial policy support and M&A premium

Analysis of the factors influencing M&A premiums is critical to understanding the operation of the M&A market and providing insight into the negotiation process [47]. Understanding how policies affect M&A premiums can help make more effective policy and corporate strategic decisions. In addition, this study provides a perspective on cross-border mergers and acquisitions and international market competitiveness, deepening the understanding of the competitive attractiveness of countries. As shown in Table 6, in model (1), industrial policy support significantly increases the M&A premium, with a coefficient of 1.708 and a significance level of ***. Model (2) holds this finding even after accounting for other variables, with a coefficient of 0.902 and a significance level of 1%. This suggests that industrial policy support remains a key factor influencing M&A premiums, even when other possible effects are considered, which is consistent with hypothesis 3. In models (3) and (4), the introduction of individual fixed effects significantly increases R2 values, reaching 0.203 and 0.322, respectively, highlighting the key role of controlling specific effects of major M&A firms in explaining changes in M&A premiums. The study also found that larger companies tend to attract higher M&A premiums, with a coefficient of 0.901 and a significance level of 1%. Other variables such as return on assets (ROA), dual attributes and sales growth rate also significantly affect the M&A premium. Refer to Table 6 for detailed coefficients and significance levels.

**Table 6:** Analysis of industrial policy support and M&A premium for target companies.

	(1) Premium	(2) Premium	(3) Premium	(4) Premium
Policy	1.708***	0.902***	2.079***	2.104***
	-4.356	-5.779	-2.322	-2.741
Size		0.901***	-0.699***	
		-3.186	-3.050	
AD		-0.015	1.892***	
		(-1.394)	-37.740	
ROA		0.269***	0.153***	
		-171	-217	
Dual		-0.025***	-0.015***	
		(-4.609)	(-2.285)	
IS		0.00	-0.021***	
		(-1.086)	(-2.712)	
SG		0.000**	-0.000*	0.000*
		(-3.787)	(-1.801)	(-2.098)
SR		-0.050***	0.081	0.043
		(-242)	-0.313	-0.474
FA		-0.001***	0.210	0.012
		(-4.016)	-2.454	-0.825
ROE		-0.062**	-0.649	-0.033
		(-1.359)	(-2.043)	(-1.499)
CG		0.003***	0.001**	0.001**
		-3.002	-3.692	-2.679
PE		0.000	0.000	0.000
		-0.015	(-0.010)	(-0.010)
Effect of individual fixation	NO	NO	YES	YES
The Effect of Time Fixation	NO	NO	YES	YES
_cons	0.142***	-0.038	0.053***	-0.438
	-23.467	(-1.072)	-1471	(-1.126)
R2	0.002	0.004	0.203	0.322
N	24362	22408	25208	20421

In models (3) and (4), the introduction of individual fixed effects significantly increases R2 values to 0.271 and 0.248, respectively, highlighting the key role of controlling specific effects of major M&A firms in explaining changes in M&A premiums. The study also found that larger companies tend to attract higher M&A premiums, with a coefficient of 0.751 and a significance level of 1%. Other variables such as return on assets (ROA), dual attributes and sales growth rate also significantly affect the M&A premium. Refer to Table 6 for detailed coefficients and significance levels.

### 5.3 Robustness analysis

In this study, the propensity score matching method is used for sample selection, re-selecting the sample and conducting robustness analysis to increase the credibility of the study [55]. The propensity score matching method effectively reduces confounding factors and increases the reliability of the results by matching the cross-border M&A sample with the non-cross-border M&A sample.

Table 7 presents an analysis using the propensity score matching technique. Here, the policy variable’s coefficient is 0.044, with a significance level of 1%, suggesting a positive correlation between the policy and the outcome variable. In the case of cmba, the scale’s coefficient is 0.010, also significant at the 1% level, indicating a positive link between company size and the outcome variables. However, in model (3) cmba, the asset-liability ratio’s coefficient is -0.003, but lacks statistical significance, implying no substantial connection between the asset-liability ratio and the outcome variable. In model (4) cmba, the return on total assets has a coefficient of 0.678 with a 1% significance level (***), affirming a positive association between total asset returns and the outcome variable. Other variables like "double", "ln", "sales growth", "wage ratio", "age", "roe", "costs", and "pe" also factor into the sales analysis.

**Table 7:** Regression results with baseline adjustment.

	(1) CMBA	(2) CMBA	(3) CMBA	(4) CMBA
Policy	0.037***	0.045***	0.022***	0.020***
	-10.237	-4.864	-1.547	-2.465
Size		0.012***		0.023***
		-2.637		-2.551
AD		-0.003		0.131***
		(-0.168)		-4.120
ROA		0.536***		0.655***
		-6.975		-157
Dual		-0.061***		-0.031***
		(-11.868)		(-1.926)
IS		-0.007*		-0.019**
		(-1.608)		(-3.148)
SG		-0.001		0.001
		(-0.285)		-0.305
SR		0.049**		0.025
		-2.985		-0.793
FA		0.000		0.027
		(-1.152)		-0.904
ROE		-0.190***		-0.082
		(-2.157)		(-1.231)
CG		0.003		0.001
		-1.082		-0.262
PE		0.000**		0.000**
		-2.005		-1.722
Individual fixation effect	NO	NO	YES	YES
Time fixation effect	NO	NO	YES	YES
_cons	0.073***	0.042	0.071***	-0.684
	(2460)	(0.648)	(13.716)	(-1.461)
R2	0.005	0.016	0.172	0.247
N	13758	14640	12648	12336

z data enclosed in brackets

* p < 0.1

** p < 0.05

*** p < 0.01

The annual adjusted regression results are shown in Table 8. For model 1, the coefficient of the policy variable is 0.092*, which reaches statistical significance at the 10% level. In model 2, the coefficient rises to 0.042***, and its significance is confirmed at the 5% level. In Model 3, by introducing individual fixed effects and considering individual differences, the impact of policies on target variables can be evaluated more accurately. Among them, the coefficient of the policy variable is 0.082*, which is significant at the 10% level. Model 4 further incorporates the time fixed effect to explain the time change, revealing that the coefficient of the policy variable is 0.012**, which is significant at the 5% level.

**Table 8:** Regression results with annual adjustments.

	(1) CMBA	(2) CMBA	(3) CMBA	(4) CMBA
Policy	0.092*	0.042***	0.082*	0.012**
	-1.478	-1.823	-1.593	-1.635
Size		0.087***		0.093***
		-5.599		-2.136
AD		0.226***		0.342***
		-160.600		-3.234
ROA		-2.234***		-1.320***
		(-1317)		(-391)
Dual		0.513***		0.353***
		-34.223		-12.098
IS		-0.054***		-0.055*
		(-3.688)		(-1.933)
SG		0		-0.003
		(-1.867)		(-0.543)
SR		1.559***		1.063***
		-20.477		-7.679
FA		-0.006***		0.153
		(-3.815)		-0.720
ROE		-0.237***		-0.222
		(-3.255)		(-1.240)
CG		0.001		0.003
		-1.256		-0.299
PE		0		0
		-0.287		(-0.099)
Effect of individual fixation	NO	NO	YES	YES
Time fixation effect	NO	NO	YES	YES
_cons	0.483***	-0.539***	0.403***	-2.577
	(39.068)	(-3.960)	(1514)	(-1.175)
R2	0.001	0.381	0.502	0.946
N	2684	3048	2367	3008

z data enclosed in brackets

* p < 0.1

** p < 0.05

*** p < 0.01

The outcomes indicate that industrial policy positively influences the decisions of Chinese companies regarding cross-border mergers and acquisitions, both in baseline and annual adjustments. Cross-border mergers and acquisitions effectively solve the needs of enterprises to expand international business and improve competitiveness [50]. This highlights the urgent need for the government to continuously improve and strengthen industrial policies. Additionally, cross-border mergers and acquisitions could play a constructive role in advancing initiatives like the Belt and Road Initiative. Industrial policy can further play its role in supporting the strategic deployment of "going global". By using propensity score matching method for sample selection and conducting robustness analysis, the findings are more credible and reliable, and the potential interference of endogeneity issues is excluded.

## 6 Conclusions, limitations and prospects of the study

### 6.1 Conclusions

An in-depth examination of this study reveals a significant positive correlation between more liberal and supportive industrial policies and cross-border M&A. These findings highlight the important role of government-led initiatives and policy actions in attracting foreign investment and facilitating cross-border M&As. This is consistent with previous studies, such as Gulen and Ion (2016), who point out the profound impact of host country economic policies on firms’ decisions and economic outcomes. In particular, industrial policies that relax financial constraints enhance firms’ ability to raise capital, thereby positively encouraging cross-border M&A firms. In addition, it was found that in China, state-owned enterprises (SOEs) have significant political affiliations in international M&As and play an important role in supporting industrial policy choices. Du and others have also noted that SOEs have an advantage over Chinese private firms in cross-border M&As. Previous research on the different mechanisms and impacts of Chinese industrial policy on cross-border M&As is limited, so this study makes a valuable contribution to filling this research gap with far-reaching implications.

The findings emphasize the importance for firms to thoroughly assess the production environment and political relations when formulating cross-border M&A strategies. In addition, this study provides important policy guidance for firms and governments in making strategic decisions in cross-border M&As. By strengthening the link between industrial policy and firms’ needs, governments can formulate policies that are more in line with market demand and firms’ growth objectives.

### 6.2 Limitations and prospects of the study

Research on the relationship between industrial policy and cross-border M&As in China relies heavily on the availability and quality of data. Obtaining reliable, accurate and comprehensive data is a challenge, especially when accessing government policy documents and detailed information on cross-border M&As. Second, the relationship between industrial policy and firm performance assessment may be endogenous. This means that policy implementation may be influenced by CBM&A activities and vice versa. This endogeneity issue increases the likelihood of biased estimates. Moreover, establishing the causal relationship between industrial policy and firm performance assessment is complex. Although panel data and time series analysis can be utilized to investigate their correlation, other variables that may affect the results, such as economic fluctuations and market demand, must be carefully considered.

In order to overcome these limitations, subsequent research should explore them in several directions. First, there is a need to obtain more accurate and detailed data, including policy documents, implementation details, and specific information about CBM&As. Second, the use of quantitative and qualitative research techniques can lead to a more comprehensive understanding of the topic. Future research could also incorporate time and regional factors to better understand the relationship between policy and MNC M&As. In addition, comparative studies of different countries or regions can provide valuable insights into the Chinese context. Case studies on specific industries or firms can also provide insights into the link between policies and M&A activities. Finally, exploring the mediating mechanisms and ways in which industrial policy affects firm performance evaluation can help to understand the relationship between the two more closely. In conclusion, although this study has provided important insights into the link between industrial policy and international M&A in China, it is important to recognize the inherent limitations in the research methodology.

Future research efforts should endeavor to address these limitations by improving data quality, addressing endogeneity issues, employing more sophisticated research methods, and considering time and regional differences. Through these studies, we can better understand the intricate link between industrial policy and firm performance assessment.

## Supporting information

S1 Data (XLS)

S2 Data (XLS)

S3 Data (XLS)

S4 Data (XLS)
